# Human satellite cells: identification on human muscle fibres

**DOI:** 10.1371/currents.RRN1294

**Published:** 2012-01-19

**Authors:** Luisa Boldrin, Jennifer E Morgan

**Affiliations:** The Dubowitz Neuromuscular Centre UCL Institute of Child Health 30 Guilford Street London WC1N 1EH United Kingdom

## Abstract

Satellite cells, normally quiescent underneath the myofibre basal lamina, are skeletal muscle stem cells responsible for postnatal muscle growth, repair and regeneration. Since their scarcity and small size have limited study on transverse muscle sections, techniques to isolate individual myofibres, bearing their attendant satellite cells, were developed. Studies on mouse myofibres have generated much information on satellite cells, but the limited availability and small size of human muscle biopsies have hampered equivalent studies of satellite cells on human myofibres. Here, we identified satellite cells on fragments of human and mouse myofibres, using a method applicable to small muscle biopsies.

## **Introduction**

Satellite cells are the principal stem cells of skeletal muscle, responsible for its homeostasis, maintenance, growth and regeneration through adulthood [1]. Satellite cells are located in a niche between the basal lamina and sarcolemma of muscle fibres [2]. Their rarity and small size are limitations for studying them on transverse muscle sections; therefore techniques were developed to prepare isolated muscle fibres, bearing their attendant satellite cells, from rodent muscles [3] [4]. These isolated, viable muscle fibres, particularly when prepared from genetically-modified mice in which either myonuclei, or satellite cells, express a reporter gene [5] [6] [7], have provided a model that has facilitated discovery of novel satellite cell markers, factors controlling satellite cell quiescence, activation, proliferation, differentiation, self-renewal and determination of their in vivo regenerative capacity [8].   

Quiescent satellite cells on isolated mouse muscle fibres express Pax7 [9], M-cadherin [10], CD34, Myf5 [11] caveolin-1 [12] [13], calcitonin receptor [13] [14], nestin [15] and α7-integrin [13] [16]. During activation, myogenic regulatory factors, such as MyoD and myogenin, are expressed, and satellite cells either self-renew or differentiate [8] [17] [18] [19] [20]. However, both in vivo and in vitro studies have demonstrated that the population of mouse satellite cells is heterogeneous, some being more “stem cell” like (i.e. able to regenerate skeletal muscle and functionally reconstitute the satellite cell compartment) than others [21] [22] [23] [24]  (reviewed in [8]).

Unfortunately, due to the difficulty in obtaining human muscle in a size and state adequate to allow the preparation of viable fibres, there is a gap in our knowledge of human, compared to mouse, satellite cells (reviewed in [8]). As a prelude to studies on quiescent human satellite cells, we wished to determine antibodies that can be reliably used for their identification. To do this, we exploited a technique to isolate fragments of human fibres from muscle biopsies of a normal individual and a Duchenne muscular dystrophy (DMD) patient. As controls, myofibre fragments from mouse muscles were prepared by the same method. We show that Pax7 and M-cadherin are reliable markers of not only mouse, but also human, satellite cells. In contrast, human satellite cells are not stained by an antibody to CD34 that recognizes mouse satellite cells and only a subpopulation of human, whereas all mouse, satellite cells express caveolin-1 [13]. Conversely, an antibody specific to human β1 integrin (CD29) identifies human, but not mouse, satellite cells. We therefore conclude that Pax7 marker defines the majority of not only mouse, but also human, satellite cells. 

## **Materials and Methods**

### *Ethics*

Tissue sampling was approved by the Institutional Ethics Committee in compliance with national guidelines regarding the use of biopsy tissue for research. Patient tissues were collected after written informed consent. Mice were bred and experimental procedures were carried out in the Biological Services Unit, University College London, in accordance with the Animals (Scientific Procedures) Act 1986.

### *Isolation of human and mouse single fibre fragments*

Biopsies of paraspinal muscle of a healthy adolescent patient (female, 14 year old) and of * extensor digitorum brevis* (EDB) muscle of a DMD adolescent patient (male, 11 year old) were collected and fixed in 4% paraformaldehyde (PFA, Sigma) at room temperature for 15 minutes. After sequential washes with phosphate buffer saline (PBS, Sigma), the sample was embedded at 4^◦ ^C, first in 30% glycerol/PBS (Sigma) overnight, and then sequentially in 50%, 80% glycerol/PBS and finally absolute glycerol until the sample sank to the bottom of the vial. The sample was then kept in glycerol at 4^◦ ^ C until dissection of fibre fragments. 

Under a stereomicroscope (Leica), fine forceps were used to dissect out fragments of fibres and exclude any other tissues, e.g. connective tissue.

As a control, *tibialis anterior* (TA) muscles from 2 transgenic 3F-*nLacZ*-2E mice (female, 2 month old), in which myonuclei, but not satellite cells express the *nLacZ* reporter gene [25], were harvested, fixed and dissected in the same way as the human muscle biopsies.

### *Immunohistochemistry of single fibre fragments*

Dissected fibre fragments were washed in PBS at room temperature, permeabilized with 0.5% Triton X-100 (Sigma) for 6 minutes, blocked for 30 minutes with 10% goat serum, and incubated overnight at 4^◦^C with the following primary antibodies: mouse monoclonal anti-Pax7 (Developmental Studies Hybridoma Bank, Iowa City, IA), rabbit polyclonal anti-laminin (Sigma), mouse monoclonal lamin A/C (Vector Laboratories), rabbit polyclonal caveolin-1 (Santa Cruz Biotechnology, Inc.), mouse monoclonal M-cadherin [10], rat monoclonal anti mouse CD34 (clone RAM34; PharMinogen), mouse monoclonal anti human CD29 (AbD Serotec). Secondary antibodies used were: Alexa Fluor 488-conjugated goat anti-mouse Ig (Molecular Probes) and Alexa Fluor 594-conjugated goat anti-rabbit Ig (Molecular Probes). Mouse fibre fragments were X-Gal stained as previously described [11]. Nuclei were counterstained with DAPI and mounted on slide in fluorescent mounting medium (Dako).

### *Microscopy*

Fluorescence detection, bright-field microscopy and image capturing were done using a Zeiss Axiophot microscope (Carl Zeiss), and Metamorph software (Metamorph Productions). 

### *Data Analysis*

Fibre length was calculated by using SigmaScan software in order to allow satellite cell quantification per 100µm fibre length. 

## **Results**

### **A high number of satellite cells is present in human fibres**

All nuclei (both myonuclei and satellite cell nuclei) on isolated fibres harvested from normal human paraspinal muscle biopsies expressed, as expected, human-specific lamin A/C in their nuclear membrane [26] [27], confirming that the fixation and fibre preparation did not interfere with immunostaining in human fibres (Figure 1A-I). 

Pax7 antibody clearly marked both human (Figure 1A II –III and Figure 2B) and mouse (Figure 2A) satellite cells; co-staining with laminin antibody confirmed that satellite cells were retained under the basal lamina of human fibres following the preparation procedure (Figure 1A- III). The mean number of human Pax7+ve satellite cells per 100µm length of fibre was 0.9 (±0.05 SEM) (Figure 1B). Considering that the paraspinal muscle is composed of fascicles of different lengths, [28] it not possible to determine the number of satellite cells per fibre. However, knowing that the mean number of satellite cells per TA myofibre is 10 [21] and that the mean length of a TA myofibre is 15mm [29], we conclude that the density of satellite cells per fibre length is higher in human paraspinal muscles than in mouse TA muscles.  



**Figure:**
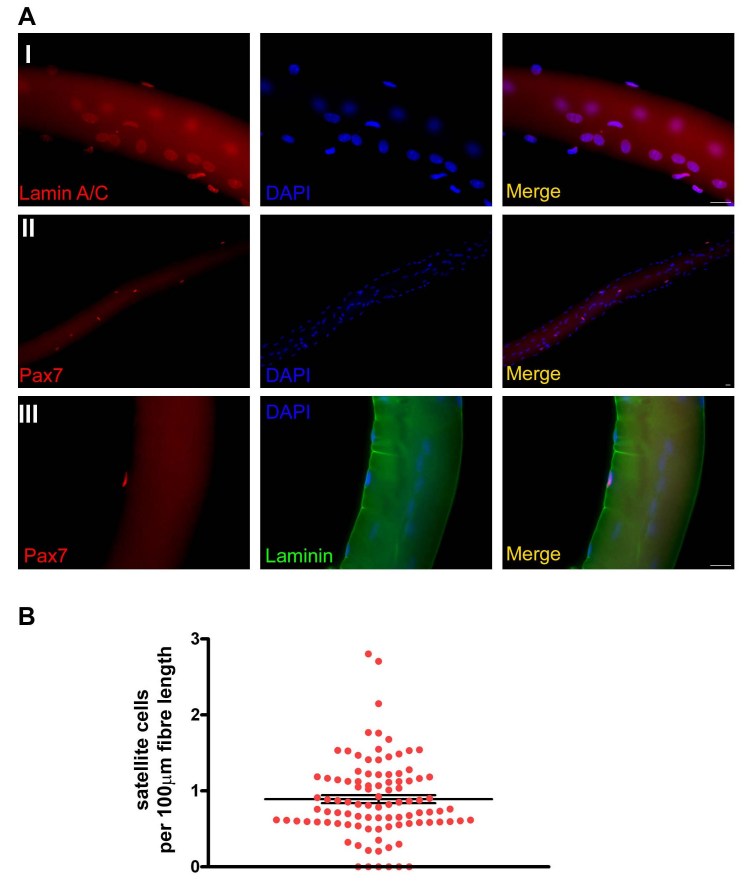
**Figure 1**

*Identification and quantification of human satellite cells on single fibres*.

All the nuclei of fragments of human myofibres of a parispinal muscle biopsy of a healthy adolescent patient were recognized by the human specific lamin A/C antibody (A I). Along the fibres, several nuclei were detected by Pax7 antibody (A II) and were in the classical satellite cell position beneath the basal lamina (A III). Nuclei were counterstained with DAPI and merged pictures are shown in panels (A I, II, III). (B) Number of satellite cells per 100µm fibre length was calculated. Scale bars: 10µm.

### **Human and mouse satellite cells are defined by a different marker signature**

As Pax7 characterizes the majority of satellite cells in mouse [9], and appears to be frequently expressed on human fibres (Figure 1A), we wished to determine if human satellite cells are identified by antibodies to other proteins expressed by mouse satellite cells. Caveolin-1, a surface protein expressed by virtually all mouse satellite cells [13] (Figure 2A), was also expressed on a sub-population of human satellite cells (Figure 2B). M-cadherin was expressed on the cell membrane of both mouse and human satellite cells (Figure 2B). We confirmed that CD34, that was reported to be expressed by mouse satellite cells [11], was indeed expressed on mouse satellite cells under the basal lamina (Figure 2E), but this antibody did not stain human satellite cells, possibly because of differences between the murine and human epitopes. Conversely, the human CD29 antibody recognised human, but not mouse satellite cells (Figure 2F). 



**Figure:**
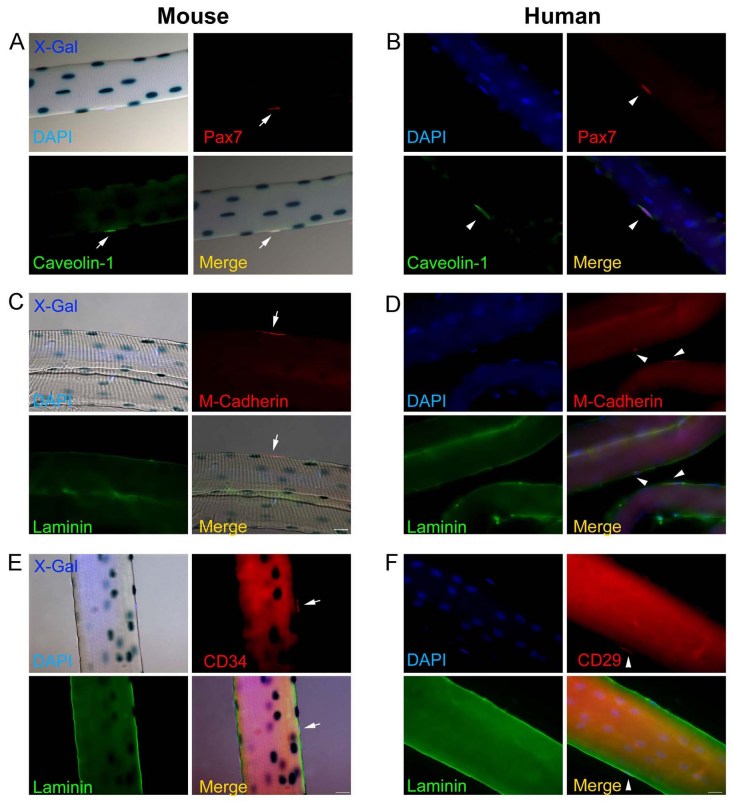
**Figure 2**

*Characterization of mouse and human satellite cells on single fibres*.

Detection of mouse (arrows) and human (arrowheads) satellite cells on single fibres. Myofibre fragments of adult 3F-*nLacZ*-2E mouse *tibialis anterior* muscle were used in order to clearly distinguish DAPI positive satellite cells from X-Gal positive myonuclei. Pax7 antibody recognizes the majority of satellite cells on both mouse (A) and human (B) fibre fragments. Caveolin-1 is expressed both in mouse (A) and human (B) satellite cells. M-Cadherin is expressed in the membrane of both mouse (C) and human (D) satellite cells. The antibody raised to mouse CD34 indeed labels only mouse satellite cells (E), whilst CD29 is a human specific antibody that reacts only with human satellite cells (F). Laminin immunostaining was performed in combination with satellite cell markers wherever possible and DAPI was used for counterstaining mouse satellite cells and human nuclei. Scale bars: 10µm.

### **Fragility of DMD myofibres does not allow dissection of single fibres**

We were not able to prepare fibre fragments derived from DMD muscles using the method of myofibre isolation that we successfully applied to normal human muscle (Figure 3A). The fragility of fibres from DMD muscle caused them to hyper-contract before the isolation and to break during the isolation process. Furthermore, the interference of connective tissue prevented single fibre isolation and only rarely could bundles of fibres be isolated for immunostaining (Figure 3B and 3C). Although our technique did not allow a clear identification of human satellite cells, Pax7 is expressed by a few nuclei in this preparation of DMD muscle (Figure 3B and C).



**Figure:**
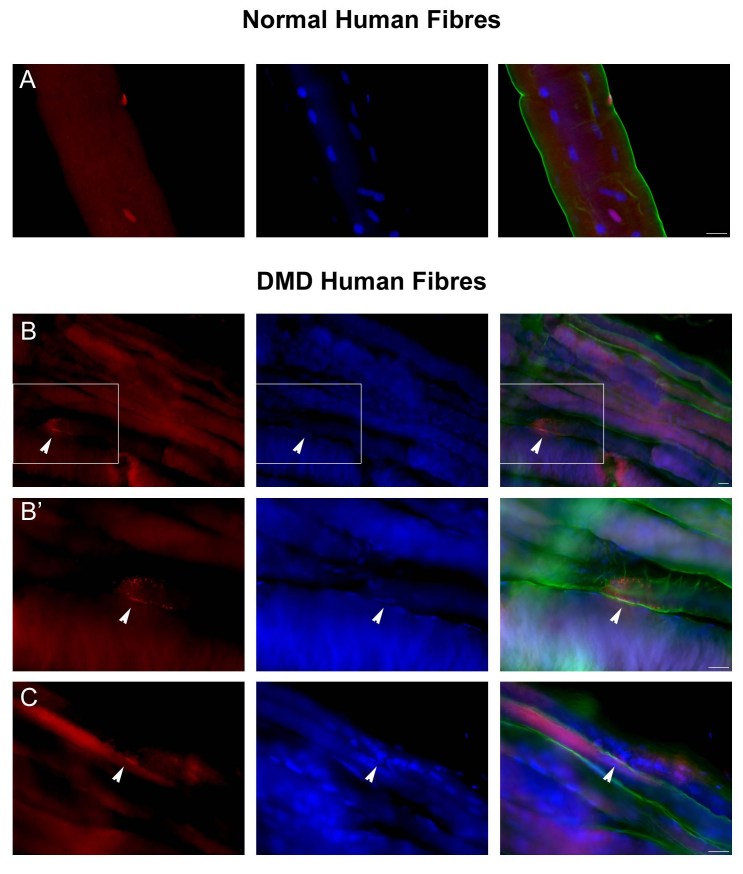
**Figure 3**

*Identification of human satellite cells on dystrophic myofibres*.

Fibre dissection was attempted from a DMD *extensor digitorum brevis* muscle. Whilst Pax7 immunostaining clearly allows satellite cell identification on healthy human single fibres below the basal lamina (A), identification is difficult and ambiguous in DMD fibres (B) even at high magnification (B’, C).

## **Discussion**

Studies on human single myofibres can be performed when muscles can be harvested from tendon to tendon, which is possible for very small muscles like abdominal, intercostals and gluteux maximus [30] [31]. However, so far, no satellite cell marker characterization has performed on such freshly-isolated human fibres. As muscle biopsies for diagnosis are usually taken from muscles such as quadriceps and biceps and as these are usually very small pieces of muscle, we wished to obtain preparations of muscle biopsies in which we could unequivocally identify satellite cells under the basal lamina of muscle fibres. The technique we have described is a suitable method to characterize human satellite cells in their quiescent state underneath the basal lamina.    

To date, most of our knowledge about human satellite cells derives from immunostaining muscle cryosections [8] [32]. However, it is challenging to distinguish on a transverse muscle section a satellite cell from an interstitial cell, or a myonucleus, particularly as both of these have been reported to occasionally express Pax7 [33]. This has caused confusion in satellite cell quantification and characterization, giving rise to contrasting reports [8].   

Mouse studies have shown that satellite cells express a mesenchymal marker, the integrin CD 29 [34]. Our study shows that human satellite cells also express CD29. However, the antibody we used against CD29 identifies human, but not mouse, satellite cells. This particular antibody might be a useful marker for detecting donor satellite cells in human/mouse xenografts.   

We cannot exclude the possibility that satellite cells in different human muscles are characterized by different markers and future investigations are needed in order to establish whether satellite cells in human muscles are heterogeneous, as has been shown in mouse  [6] [35].    

Although the technique that we used was not applicable to DMD muscles, we suspect that, were a DMD biopsy available in the early stage of the pathology, when fibres are not so fragile, we would be able to obtain fibre fragments for study. Moreover, for disorders such as polymyositis, DMD and myotonic dystrophy, where satellite cell number is known to be altered [36], quantification of satellite cells would be informative.   

In summary, we have shown that a clear qualitative and quantitative identification of satellite cells can be performed by analysis of human muscle fibre fragments. Further investigations are needed in order to explore the cell biology of human satellite cells.  

### Corresponding author: 

Luisa Boldrin, UCL, Institute of Child Health, 30 Guilford Street, London WC1N 1EH, United Kingdom. 

## **Acknowledgements**

We would like to thank the Biobank of the MRC Centre for Neuromuscular Diseases that provides human muscle cells and tissue for research. We would also like to thank Dr Geraldine Edge, Department of Anaesthetics, Stanmore Hospital, London and Dr Jan Lehovsky, Department of Orthopaedics, Stanmore Hospital, London for providing us with human muscle samples. The Pax7 antibody developed by A. Kawakami was obtained from the Developmental Studies Hybridoma Bank developed under the auspices of the NICHD and maintained by the University of Iowa, Department of Biological Sciences, Iowa City, IA 52242.

## **Funding information**

LB is founded by the Muscular Dystrophy Campaign (MDC) and JM holds a Wellcome Trust University award.

## **Competing interests**

The authors have declared that no competing interests exist.
